# Determining the quality of educational climate across multiple undergraduate teaching sites using the DREEM inventory

**DOI:** 10.1186/1472-6920-5-8

**Published:** 2005-02-21

**Authors:** Rajesh Varma, Ekta Tiyagi, Janesh K Gupta

**Affiliations:** 1 Academic Department of Obstetrics and Gynaecology, Birmingham Women's Hospital, Birmingham, B15 2TG, UK

## Abstract

**Background:**

Our obstetrics and gynaecology undergraduate teaching module allocates 40–50 final year medical students to eight teaching hospital sites in the West Midlands region. Based on student feedback and concerns relating to the impact of new curriculum changes, we wished to objectively assess whether the educational environment perceived by students varied at different teaching hospital centres, and whether the environment was at an acceptable standard.

**Methods:**

A Dundee Ready Education Environment (DREEM) Questionnaire, a measure of educational environment, was administered to 206 students immediately following completion of the teaching module.

**Results:**

The overall mean DREEM score was 139/200 (70%). There were no differences in the education climate between the teaching centres.

**Conclusion:**

Further research on the use of DREEM inventory, with follow up surveys, may be useful for educators to ensure and maintain high quality educational environments despite students being placed at different teaching centres.

## Background

The undergraduate curriculum at our medical school was redesigned in 1998/99 to bring it in line with recommendations suggested by the General Medical Council (GMC) in Tomorrow's Doctors [1]. Obstetrics and Gynaecology is taught as a final year module. Around 20–30 students, of a total year group of around 200 students, are allocated to eight teaching hospital sites in the West Midlands region, and remain their for the length of the module (eight weeks). Throughout the placement, all formal lectures take place at the principal Teaching Hospital (Birmingham Women's Hospital). A comprehensive course handbook and web-based multiple choice formative assessment accompany the module, and detail the teaching, practical and assessment objectives for students and clinicians. We have aimed to ensure there are no significant differences in the way the curriculum is delivered between centres. All 200 students sit the final exam in Obstetrics and Gynaecology straight after completing the 8-week course module.

Based on previous student feedback reporting differences in educational experiences, together with our concerns relating to the impact of new curriculum changes, we wished to objectively assess whether the educational environment perceived by students varied at different teaching hospital centres, and whether the environment was at an acceptable standard. In particular, was there any potential loss of teaching experience when students were placed away from the principal Teaching Hospital. Thus, the null hypothesis we wished to test was that there was no difference in the learning environment between centres. Several questionnaire-based educational tools are available that set out to 'quantify' the educational environment [2-4]. However, we chose to use the Dundee Ready Education Environment Measure (DREEM) inventory, as more studies had evaluated and validated this method [5,6]. The DREEM inventory consists of 50 questions, each scoring 4, giving a total maximum individual DREEM score of 200. The five domains that comprise the DREEM are depicted in Table 1.

**Table 1:** Domains of DREEM questionnaire

**TOPIC**	**Number of questions**	**Maximum DREEM Score**
Students' Perception of Learning	12	48
Students' Perception of Teachers	11	44
Students' Academic Self-Perceptions	8	32
Students' Perception of Atmosphere	12	48
Students' Social Self-Perceptions	7	28
**Total**	**50**	**200**

## Methods

The DREEM questionnaire, based on a Likert scale, was administered to the full class of 206 final-year Birmingham University medical students undertaking the exam module in Obstetrics and Gynaecology in 2000. All questionnaires were distributed and returned the same day of the exam, which allowed us to achieve a 100% response rate. Students were told to only comment on their recent 8-weeks experience of Obstetrics and Gynaecology. Statistical analysis was performed using Microsoft Excel and Arcus Quickstat Biomedical Statistical software, and utilised single-sample T test and One-way analysis of variance (ANOVA).

## Results

**Figure 1:**
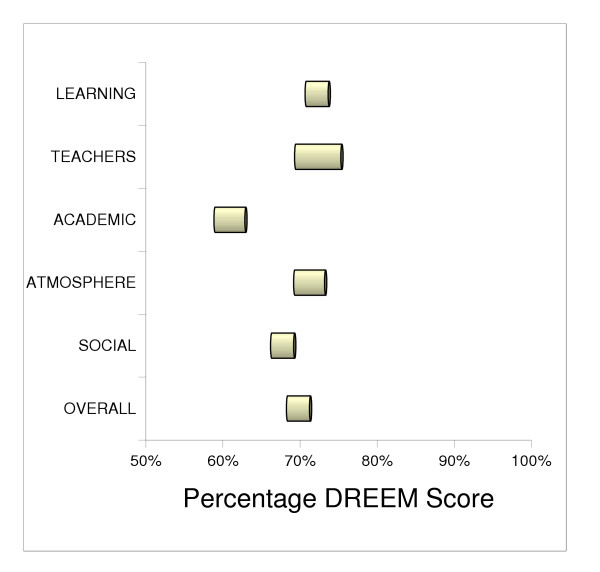
Graphical representation of the contribution of each DREEM domain to the overall mean DREEM score

The year group comprised 42% male and 58% female. The overall mean DREEM score for the study group was 139/200 (95% CL 136.1 to 141.9), or expressed as percentage of the maximal score, 70% (95% CL 68% to 71%). There was no statistically significant difference between the mean scores for the contributory DREEM domains, which were as follows: perception of learning, 34.52/48 (72%); perception of teaching, 32.05/44 (73%); academic self-perception, 19.46/32 (61%); perception of atmosphere, 34.07/48 (71%), and for social self perceptions, 18.90/28 (68%). The DREEM scores for each hospital, with comparison of all contributory elements of the DREEM inventory, are depicted in Table 2 and Figure 1.

**Table 2:** The DREEM domains and overall score for each hospital

HOSPITAL	Number of Students	LEARNING Mean Score/48		TEACHERS Mean Score/44		ACADEMIC SELF-PERCEPTION Mean Score/32		ATMOSPHERE Mean Score/48		SOCIAL Mean Score/28		OVERALL DREEM Score/200		DREEM percentage for each hospital
	(total of 206)	Percentage of maximum score		Percentage of maximum score		Percentage of maximum score		Percentage of maximum score		Percentage of maximum score		Percentage of maximum score		
BWH	53	33.77		31.89		19.77		33.40		19.32		138.15		69%
Good Hope	20	33.30		30.10		18.15		33.40		18.20		133.15		67%
B'ham Heartlands	26	34.15		32.73		18.92		32.77		19.58		138.15		69%
Walsall Manor	20	34.10		34.35		19.90		34.30		19.05		141.70		71%
City	32	35.13		28.31		19.41		34.97		17.75		135.56		68%
Wolverhampton	22	35.77		32.59		20.41		33.64		18.86		141.27		71%
Shrewsbury	13	34.77		32.85		18.69		35.00		19.15		140.46		70%
Wordsley	20	35.15		33.60		20.40		35.10		19.30		143.55		72%
														
Mean overall		34.52	72%	32.05	73%	19.46	61%	34.07	71%	18.90	68%	139.00	70%	
Lower 95% CL		33.83	70%	30.41	69%	18.77	59%	33.33	69%	18.38	66%	136.13	68%	
Upper 95% CL		35.21	73%	33.69	77%	20.14	63%	34.82	73%	19.42	69%	141.88	71%	

CL Confidence Limit

When converting the raw DREEM score to percentages, two-sided P-value single-sample Student's T test showed no statistically significant difference between hospitals by each DREEM domain, or between each DREEM domain within the same hospital. Greatest variation between hospitals occurred in the Students' Perception of Atmosphere domain, where there were four hospitals beyond the 95% Confidence Limits; this compared to three hospitals beyond 95% Confidence Limits in all other DREEM domains (Table s2). One-Way analysis of variance (ANOVA) yielded F (variance ratio) = 0.5222, P = 0.8111, which indicated no statistically significant differences between hospitals, DREEM domains, or overall DREEM scores (Table 3).

**Table 3:** ANOVA analysis between different hospitals for the differing DREEM domains

	Percentage of maximum score for each DREEM component for each hospital							
	Birmingham Women's	Good Hope	Birmingham Heartlands	Walsall	City	Wolver-hampton	Shrewsbury	Wordsley

Learning	70%	69%	71%	71%	73%	75%	72%	73%
Teachers	72%	68%	74%	78%	64%	74%	75%	76%
Academic	62%	57%	59%	62%	61%	64%	58%	64%
Atmosphere	70%	70%	68%	71%	73%	70%	73%	73%
Social	69%	65%	70%	68%	63%	67%	68%	69%

One-way analysis of variance (ANOVA) yielded F (variance ratio) = 0.5222, P = 0.8111.

## Discussion

We have used the Dundee Ready Education Environment Measure (DREEM) in 'diagnosing' the educational environment of eight different teaching centres and making comparative analysis between these centres. The overall mean DREEM score was 139/200, or expressed as a percentage, 70% (95% CL 68–71%). The educational learning environment did not vary between centres. The two lowest scoring contributory domains, academic self-perception (61%) and social self-perceptions (68%), were not statistically significantly different from the other three DREEM domains or overall mean DREEM score.

This study has benefited by using an established educational measure and obtaining a 100% response rate. No students had been previously taught at the principal teaching hospital as this was solely used for Obstetrics and Gynaecology teaching. However, some of the students (surveyed to be 16/206, 8%) had previously attended the other seven teaching hospital centres due to prior clinical teaching attachments. Thus, previous experiences may have biased the teaching assessment completed by some students. Furthermore, the DREEM questions are of such a nature that it is likely that the environment of the entire curriculum was being assessed. However, by performing the DREEM survey immediately at the end of the obstetrics and gynaecology module, and emphasising reporting only the last eight weeks experience, we believe this maximised the chance that the DREEM measure assessed only the recent hospital teaching site and minimised any recall bias. Other groups [7] have highlighted the potential flaws in using means and parametric statistical tests on ordinal data from Likert scales. As there is no firmly established consensus, we adopted to use the Student's T test and ANOVA calculation to fulfil best statistical methodology.

The DREEM domains are unlikely to be independent variables, and may be less of an environment test but more of a measure of the overall motivation and learning attitude of the individual. The Course Valuing Inventory (CVI) score is made up of five domains: worthiness of learning experience, emotional awareness, personal development, cognitive enhancement and task drive. A recent study of first year medical students showed a correlation between higher Course Valuing Inventory (CVI) scores, female gender, stronger self-confidence as a learner, greater motivation to learn and higher DREEM scores [8].

There is no accepted agreement on what is an acceptable DREEM inventory score from published literature. Nevertheless, our DREEM score of 139/200 was higher than other reports. A study of final year medical students in Trinidad reported an overall mean DREEM of 109.9/200 [5]. A larger scale study, involving students from both final and earlier undergraduate training years, showed a DREEM score of 118/200 in a Nigerian medical school, and 130/200 in a Nepalese medical school [9]. Our higher score is reassuring, and is perhaps an indicator of better hospital teaching environment, the positive value of using a comprehensive course handbook, and the encouragement of formative self-assessment as guided by the course handbook and web-based package.

The non-significant differences between the DREEM domains and between hospitals were significant findings. This was conveyed to our tutors based at the various teaching centres as a positive and encouraging result. In practical terms, this meant that regardless of hospital capacity or student group size, their education delivery and environment was no different to other centres in the student's curriculum. The DREEM inventory may thus be a useful tool for educators to ensure and maintain high quality educational environments and uniformity in educational delivery despite students being placed at different teaching centres.

## Competing interests

The author(s) declare they have no competing interests.

## Authors' contributions

RV and ET carried out the statisitcal analysis, data interpretation, and drafted the manuscript. JKG conceived and coordinated the study, acquired the results, and made revisions of the manuscript. All authors read and approved the final manuscript.

## Pre-publication history

The pre-publication history for this paper can be accessed here:


